# Extracellular Electron Transfer Powers Enterococcus faecalis Biofilm Metabolism

**DOI:** 10.1128/mBio.00626-17

**Published:** 2018-04-10

**Authors:** Damien Keogh, Ling Ning Lam, Lucinda E. Doyle, Artur Matysik, Shruti Pavagadhi, Shivshankar Umashankar, Pui Man Low, Jennifer L. Dale, Yiyang Song, Sean Pin Ng, Chris B. Boothroyd, Gary M. Dunny, Sanjay Swarup, Rohan B. H. Williams, Enrico Marsili, Kimberly A. Kline

**Affiliations:** a Singapore Centre for Environmental Life Science Engineering, Nanyang Technological University, Singapore; b School of Biological Sciences, Nanyang Technological University, Singapore; c Interdisciplinary Graduate School, Nanyang Technological University, Singapore; d Singapore Centre for Environmental Life Science Engineering, National University of Singapore, Singapore; e Department of Microbiology, University of Minnesota Medical School, Minneapolis, Minnesota, USA; f Singapore Phenome Center, Lee Kong Chian School of Medicine, Nanyang Technological University, Singapore; g School of Materials Science and Engineering, Nanyang Technological University, Singapore; h Department of Biological Sciences, National University of Singapore, Singapore; i School of Chemical and Biomedical Engineering, Nanyang Technological University, Singapore; University of Kansas; Washington University School of Medicine

**Keywords:** Enterococcus faecalis, biofilm, extracellular electron transfer, iron, metabolism

## Abstract

Enterococci are important human commensals and significant opportunistic pathogens. Biofilm-related enterococcal infections, such as endocarditis, urinary tract infections, wound and surgical site infections, and medical device-associated infections, often become chronic upon the formation of biofilm. The biofilm matrix establishes properties that distinguish this state from free-living bacterial cells and increase tolerance to antimicrobial interventions. The metabolic versatility of the enterococci is reflected in the diversity and complexity of environments and communities in which they thrive. Understanding metabolic factors governing colonization and persistence in different host niches can reveal factors influencing the transition to biofilm pathogenicity. Here, we report a form of iron-dependent metabolism for Enterococcus faecalis where, in the absence of heme, extracellular electron transfer (EET) and increased ATP production augment biofilm growth. We observe alterations in biofilm matrix depth and composition during iron-augmented biofilm growth. We show that the *ldh* gene encoding l-lactate dehydrogenase is required for iron-augmented energy production and biofilm formation and promotes EET.

## IMPORTANCE

Bacterial metabolic versatility can often influence the outcome of host-pathogen interactions, yet causes of metabolic shifts are difficult to resolve. The bacterial biofilm matrix provides the structural and functional support that distinguishes this state from free-living bacterial cells. Here, we show that the biofilm matrix can immobilize iron, providing access to this growth-promoting resource which is otherwise inaccessible in the planktonic state. Our data show that in the absence of heme, Enterococcus faecalis l-lactate dehydrogenase promotes EET and uses matrix-associated iron to carry out EET. Therefore, the presence of iron within the biofilm matrix leads to enhanced biofilm growth.

## INTRODUCTION

Human colonization by enterococci initiates immediately at birth via gastrointestinal inoculation from maternal sources, diet, and the environment (1). Enterococcus faecalis represents a significant proportion of this early enterococcal population and remains a stable member of the community throughout life (1). In the gastrointestinal tract, enterococci are present in the lumen as well as in more specialized niches in the physicochemically complex mucus epithelial layer and epithelial crypts in the small intestine (2).

Bacterial biofilms contribute to host health and disease and are complex systems that rely on the biofilm matrix to provide the structural and functional properties that distinguish this state from free-living bacterial cells (3). The dynamics of biofilm formation are dependent on many factors such as nutrient availability, environmental stress, social competition, and the generation of extracellular matrix materials. These extracellular polymeric substances provide the architecture surrounding the bacterial cells enabling emergent properties such as resource capture, tolerance to antimicrobial compounds, cooperation or competition, and localized gradients. Biofilm-associated enterococcal infections can be especially difficult to treat due to their increased tolerance to antimicrobials and immune clearance (4).

The lactic acid bacteria (LAB), which include enterococci, use an electron transport chain for aerobic respiration when external heme is provided or, alternatively, can perform fermentation in the absence of heme. E. faecalis does not synthesize heme *de novo*, and therefore, the bacterium lacks porphyrin rings of heme required for cytochrome *bd* activity during cellular respiration (5). Similar to other members of the lactic acid bacterial group, E. faecalis has little or no requirement for nutritional iron, with manganese instead being used as the essential cofactor for cellular processes (6–9). Yet, E. faecalis undergoes transcriptional reprogramming when environmental iron availability changes (10–12), suggesting an alternative function of iron for this organism. E. faecalis is also remarkably resistant to oxidative stress from O_2_^−^, hydroxyl radicals (OH˙), and hydrogen peroxide (H_2_O_2_) sources from incomplete reduction of oxygen during respiration, from the oxidative action of host immune cells, or that arise during the Fenton reaction (13, 14). These findings suggest that E. faecalis may be able to withstand iron at concentrations which are typically toxic to most bacterial species. In human serum, free iron is far below the 10^−7^ to 10^−5^ M optimal range required to support the growth of most bacteria (15). However, iron availability in the host can vary. Disease states such as hemochromatosis and thalassemia disrupt homeostasis and cause excess iron accumulation (16–19). While there are no strong clinical links between iron overload disorders and enterococcal infection in humans, enterococcal overgrowth has been documented in the gastrointestinal tract of mice carrying mutations associated with hereditary hemochromatosis (19), suggesting that there may be physiological niches where enterococci may encounter excess iron. Moreover, iron oxides have also been detected in human spleens and the iron concentration of the gastrointestinal tract is sufficient to support bacterial growth (20, 21).

In this study, we hypothesize that the ability to withstand higher iron concentrations in some environments provides a metabolic advantage to E. faecalis. We show that iron can accumulate in the E. faecalis biofilm matrix and augments biofilm growth. Using chronocoulometry, we show that iron-augmented biofilms undergo extracellular electron transport (EET), in an *ldh-*dependent manner. Together, these data support a model in which the E. faecalis biofilm matrix harbors iron sinks which in turn promote EET, altered metabolism, and augmented biofilm growth. Understanding the metabolic factors that promote colonization and biofilm formation under different environmental conditions may inform mechanisms governing the switch from free-living planktonic to biofilm enterococci in a number of ecologic reservoirs.

## RESULTS

### Iron supplementation promotes Enterococcus faecalis biofilm growth and alters biofilm matrix and matrix properties.

**FIG 1:**
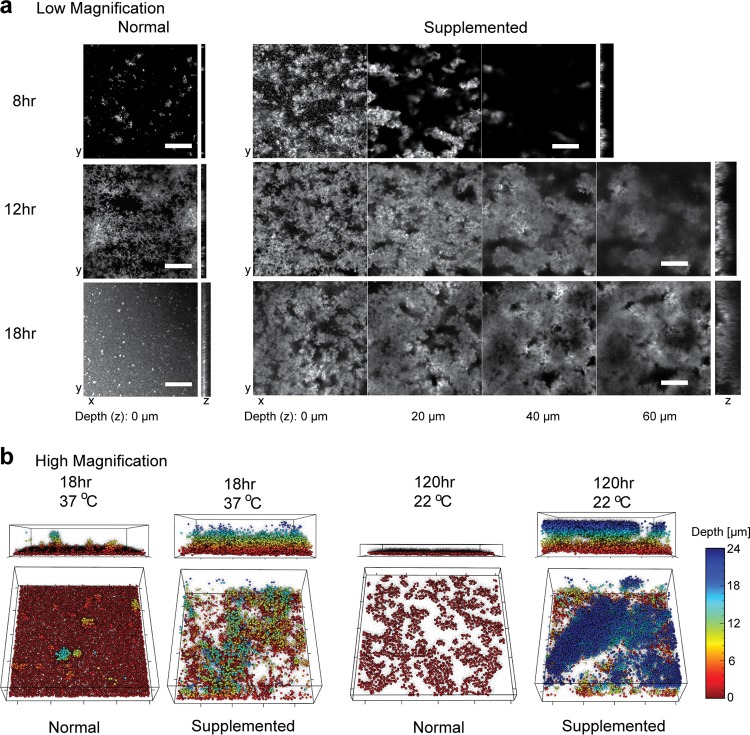
E. faecalis flow cell biofilms in iron-supplemented medium. (a) CLSM images at 8, 12, and 18 h for normal (10% TSBG) and supplemented (10% TSBG plus 0.2 mM FeCl_3_) media. Selected optical sections at the indicated depths are followed by representative lateral views in *yz* at the right of each image set. Bar, 100 μm. (b) 3D reconstructions of high-magnification CLSM stacks of E. faecalis flow cell biofilms grown in normal or supplemented medium for 18 h at 37°C or 120 h at 22°C. Biofilm depth is color coded as indicated on *z*-depth scale (0 to 24 μm), and the lateral box dimensions are 85 by 85 µm (20 µm between ticks).

**FIG 2:**
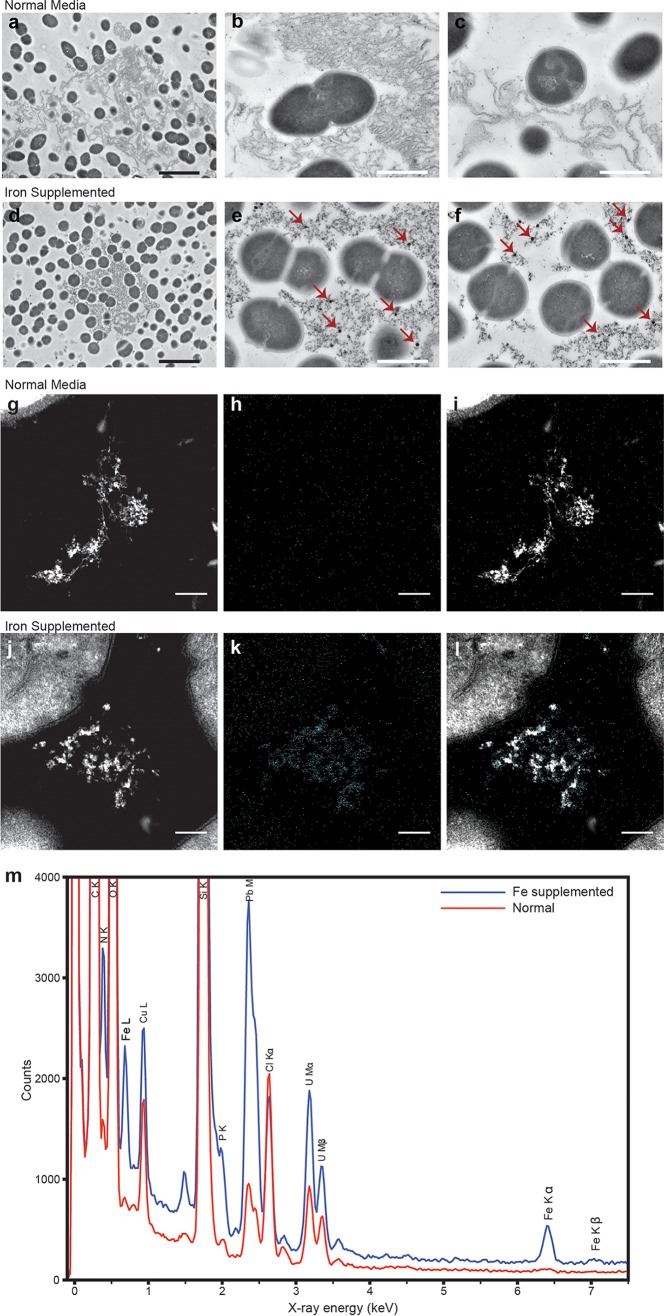
Electron micrographs of the E. faecalis biofilm matrix with iron supplementation. Representative images from TEM of E. faecalis biofilm from flow cell in normal 10% TSBG (a, b, and c) or 10% TSBG supplemented with 0.2 mM FeCl_3_ (d, e, and f). In panels a to f, bars represent 2 μm (black) and 0.5 μm (white), and red arrows highlight examples of electron-dense particles. The biofilm matrix from biofilms grown in normal medium (g, h, and i) or with iron supplementation (j, k, and l) was examined by HAADF STEM and EDS mapping at ×300,000 magnification: HAADF STEM (g and j), iron EDS map (h and k), and merged EDS-STEM images (i and l) are shown at ×300,000 magnification. Bars in panels g to l represent 1 µm. The EDS spectra for iron, corresponding to the images, are shown in panel m.

To understand the influence of iron on E. faecalis biofilm, we evaluated biofilm growth using ferric chloride (FeCl_3_)-enriched medium. We analyzed biofilm by confocal laser scanning microscopy (CLSM) in the absence (normal) and presence (supplemented) of additional FeCl_3_ in a flow cell biofilm system. Using a green fluorescent protein (GFP)-expressing E. faecalis strain grown in flow cells, we observed augmented biofilm as early as 8 h in medium supplemented with 0.2 mM FeCl_3_ compared to normal medium (Fig. 1a). Iron-enhanced biofilm growth continued for 18 h, in contrast to the thinner normal biofilm. Iron supplementation did not augment E. faecalis growth in planktonic culture (see Fig. S1 in the supplemental material), indicating that iron-mediated augmentation of E. faecalis growth is a biofilm-specific phenotype. For detailed visualization of E. faecalis cell distribution and biofilm architecture properties, we created three-dimensional (3D) reconstructions of high-magnification CLSM stacks (Fig. 1b). At 18 h, 37°C E. faecalis biofilm forms a flat monolayer in the normal medium as reported previously (22, 23). In contrast, E. faecalis biofilm is thicker and the bacterial cells are distributed throughout the biofilm when iron is supplemented. We therefore hypothesized that E. faecalis gains a metabolic advantage by the supplementation of iron, which in turn affects growth rate. We reasoned that at a reduced temperature, the E. faecalis growth rate would decrease and possibly modulate the dependence on iron supplementation. At 120 h, E. faecalis grown at 22°C forms a sparse monolayer biofilm in normal medium; however, the biofilm expands to a depth of greater than 24 μm (the image resolution limit of CLSM) and increased cell density in iron-supplemented medium. To evaluate morphological changes in E. faecalis cells or the biofilm matrix, we analyzed biofilm cells grown in flow cells by transmission electron microscopy (TEM). We observed typical diplococcal E. faecalis in the normal biofilm cells with an abundance of extracellular fibrous material in the matrix (Fig. 2a, b, and c). Constituents of the E. faecalis biofilm matrix are not well characterized; however, E. faecalis can actively secrete extracellular DNA (eDNA) in biofilms, and DNase treatment can disperse E. faecalis biofilms, suggesting that eDNA is a major component of the matrix (24–26). TEM images of the iron-supplemented biofilm are similar to normal biofilm (Fig. 2a, b, and c and S2b), with the exception of electron-dense particles associated with the extracellular fibrous matrix material (Fig. 2d, e, and f and S2f). Using energy-dispersive X-ray spectroscopy (EDS) in a TEM to analyze the same samples, we demonstrated the extracellular electron-dense particles to be enriched in iron (Fig. 2g to m and S2h and i) and hypothesize that these iron deposits, together with the increased biomass observed by CLSM, are important for augmented E. faecalis biofilm formation.

10.1128/mBio.00626-17.1 FIG S1  E. faecalis planktonic growth under metal supplementation. Time course enumeration of E. faecalis planktonic growth over 24 h in TSBG and TSBG supplemented with 2 mM FeCl_3_. *n* = 3 biological replicates. Error bars represent standard deviations from the means. Two-way analysis of variance with Bonferroni multiple-comparison test was performed, and only the 8-h time point (no Fe versus Fe) had a *P* value of <0.01 (**). Download FIG S1, TIF file, 1.9 MB. Copyright © 2018 Keogh et al. 2018 Keogh et al. This content is distributed under the terms of the Creative Commons Attribution 4.0 International license.

10.1128/mBio.00626-17.2 FIG S2  Electron micrographs and EDS of the E. faecalis biofilm matrix in the absence or presence of metal supplementation. Representative images from TEM of E. faecalis biofilm from 24-h static biofilms grown in normal TSBG (a and e), TSBG supplemented with 2 mM FeCl_3_ (b and f), or 50 µM heme (c and g). Bars, 2 μm (black) and 0.5 μm (white). The biofilm matrix from biofilms grown in normal medium (h) or with iron supplementation (i) was examined by HAADF STEM and EDS mapping at ×300,000 magnification: HAADF STEM (electron), iron EDS map (Fe), oxygen EDS map (O), lead EDS map (Pb), copper EDS map (Cu), and uranium EDS map (U) are shown at ×300,000 magnification. Scale bars in panels g to i represent 1 µm. The HAADF STEM and EDS spectra for iron are the same as shown in Fig. 2. Download FIG S2, JPG file, 1.9 MB. Copyright © 2018 Keogh et al. 2018 Keogh et al. This content is distributed under the terms of the Creative Commons Attribution 4.0 International license.

### Iron-induced Enterococcus faecalis growth is biofilm specific, and iron availability impacts the metallome.

**FIG 3:**
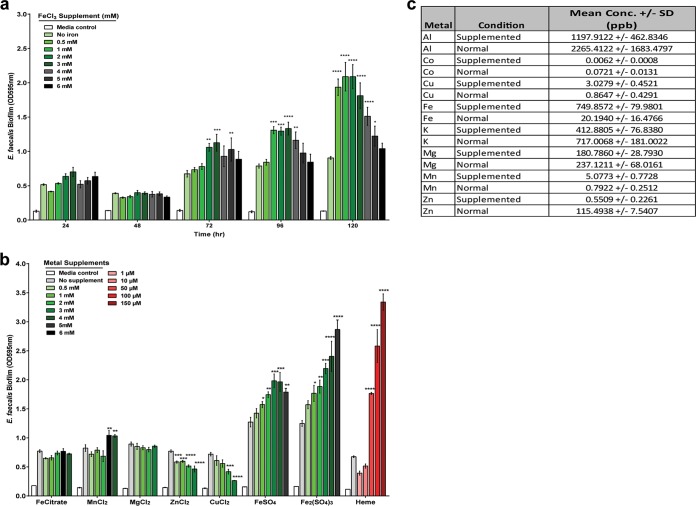
E. faecalis biofilm growth under metal supplementation. (a) Time course of E. faecalis biofilm growth in TSBG supplemented with FeCl_3_. (b) E. faecalis biofilm growth at 120 h in TSBG supplemented with metals as indicated. (c) ICP-MS analysis of E. faecalis grown in TSBG (normal) and TSBG supplemented with 2 mM FeCl_3_ (supplemented). (a and b) Data at each time point or metal supplement represent an independent experiment, with the data merged for representation. (a to c) *n* = 3 biological replicates. Statistical significance was determined by two-way analysis of variance with Tukey’s test for multiple comparisons. *n* = 3 with four technical replicates. *, *P* ≤ 0.05; **, *P* ≤ 0.01; ***, *P* ≤ 0.001; ****, *P* ≤ 0.0001. (a) Statistical analysis was calculated with time set as a repeated measure. (a, b, and c) Error bars represent standard deviations from the mean.

To determine if redox-active metals other than iron and heme could also augment E. faecalis biofilm formation, we performed static biofilm assays via crystal violet (CV) staining in microtiter plates to increase throughput (27). In contrast to continuous-feed flow cell biofilms where we use diluted (10%) medium and 0.2 mM FeCl_3_ because nutrients are not limiting, we use full-strength medium and 2 mM FeCl_3_ for microtiter plate biofilms because they are a closed-batch system where nutrients are finite (28). In all biofilm assays, we included abiotic medium controls with metals supplemented to monitor metal precipitation and found that no metals fell out of solution at the concentrations tested in these assays (data not shown). Consistent with E. faecalis biofilm augmentation observed when grown in flow cells with 0.2 mM supplemented FeCl_3_ (Fig. 1a and b), 2 mM FeCl_3_-enriched medium augmented E. faecalis static biofilm growth over time (0 to 120 h) compared to the normal medium (Fig. 3a). The biomass was significantly greater at FeCl_3_ concentrations of 0.5 to 2 mM (Fig. 3a). Above these concentrations, biomass was also enhanced but not as significantly compared to the nonsupplemented control. We next investigated the capacity of different metal species to augment E. faecalis biofilm accumulation and found that supplementation with ferrous sulfate (FeSO_4_) or ferric sulfate [Fe_2_(SO_4_)_3_] also increased E. faecalis biofilm growth, whereas ferric citrate and magnesium chloride (MnCl_2_) had negligible effects on E. faecalis biofilm (Fig. 3b). Zinc chloride (ZnCl_2_) and copper chloride (CuCl_2_) modestly diminished biofilm formation, suggesting that E. faecalis biofilm cells may be more sensitive to zinc- or copper-mediated toxicity. Manganese chloride had a modest augmenting effect on biofilm formation. Heme supplementation at concentrations from 50 to 150 µM also significantly promoted biofilm accumulation and is likely the result of enhanced activity in cytochrome *bd* since heme functions as a cofactor for this enzyme and drives E. faecalis to aerobic respiration (29).

TEM analysis (Fig. 2d, e, and f) demonstrated iron deposits in the extracellular biofilm matrix of the iron-supplemented cultures, whereas no extracellular deposits were observed for biofilms supplemented with heme (Fig. S2a to g). We therefore hypothesized that extracellular iron was promoting biofilm growth and that iron concentrations in the E. faecalis intracellular metallome would remain stable between the normal and iron-supplemented cultures. We used inductively coupled plasma mass spectrometry (ICP-MS) to quantify the intracellular metallome of E. faecalis in the absence (normal) and presence (supplemented) of FeCl_3_ supplementation (Fig. 3c). While the summed total intracellular metal abundance decreased in the supplemented medium (2,550 ppb) compared to the normal medium (3,360 ppb) (Fig. 3c), intracellular iron in the supplemented samples was greater (750 ppb) than in the normal samples (20 ppb) (Tables S1 and S2). The increase in intracellular iron concentration was coincident with a decrease in intracellular cobalt, aluminum, potassium, magnesium, and zinc when E. faecalis was grown in iron-supplemented medium. We also observed an increase in the redox-active metals copper and manganese. Together, these data suggested that iron was substituting for some metals in the supplemented medium and that the substitution was likely to be functionally balanced. Iron, cobalt, and zinc are transition metals that can function as interchangeable ions and can compete with iron for binding to metalloproteins (30). This may explain the decrease in cobalt and zinc levels. The increase in intracellular iron in the supplemented samples suggests that iron may play an intracellular role in biofilm augmentation. Together with the extracellular electron-dense deposits, iron flux across the membrane may be contributing to biofilm growth. We hypothesized that identifying genes involved in metal acquisition would help determine if intracellular iron was important for iron-enhanced biofilm growth. We examined 21 mutants of uncharacterized E. faecalis membrane transport or regulation systems, with homology to previously characterized metal-associated proteins in other bacteria, and measured intracellular metal content by ICP-MS (Tables S1, S2, and S3) (31). However, these mutants did not have significant changes in intracellular iron in the normal or supplemented medium. Because bacterial iron transport systems are generally expressed under iron limitation, we also tested these mutants in an iron-chelated medium where E. faecalis planktonic growth has previously been shown to be growth limited (10) and found that 15 mutants were attenuated for iron uptake when iron was limiting (Table S4). Overall, the ICP-MS analysis has demonstrated modulation of the E. faecalis metallome when cultured at different iron concentrations and identified systems involved in metal acquisition. However, this study did not identify any systems involved in iron acquisition in the supplemented medium, suggesting that they are not involved or that there is a large amount of overlapping functionality in iron transport in E. faecalis.

10.1128/mBio.00626-17.6 TABLE S1  Metallome comparison of E. faecalis mutants versus wild type in normal medium. Download TABLE S1, XLSX file, 0.02 MB. Copyright © 2018 Keogh et al. 2018 Keogh et al. This content is distributed under the terms of the Creative Commons Attribution 4.0 International license.

10.1128/mBio.00626-17.7 TABLE S2  Metallome comparison of E. faecalis mutants versus wild type in supplemented medium. Download TABLE S2, XLSX file, 0.02 MB. Copyright © 2018 Keogh et al. 2018 Keogh et al. This content is distributed under the terms of the Creative Commons Attribution 4.0 International license.

10.1128/mBio.00626-17.8 TABLE S3  Metallome comparison of E. faecalis mutants versus wild type in depleted medium. Download TABLE S3, XLSX file, 0.02 MB. Copyright © 2018 Keogh et al. 2018 Keogh et al. This content is distributed under the terms of the Creative Commons Attribution 4.0 International license.

10.1128/mBio.00626-17.9 TABLE S4  E. faecalis mutants in iron-depleted medium where iron was not detected. Download TABLE S4, XLSX file, 0.01 MB. Copyright © 2018 Keogh et al. 2018 Keogh et al. This content is distributed under the terms of the Creative Commons Attribution 4.0 International license.

### Genes involved in E. faecalis metabolism contribute to iron-induced biofilm growth.

To determine the mechanism underlying iron-enhanced E. faecalis biofilm, we screened a near-saturated E. faecalis mariner transposon (Tn) library for changes in biofilm formation in medium supplemented with 2 mM FeCl_3_ (31). Using the CV biofilm assay, we screened the transposon library for mutants displaying either loss or further enhancement of biofilm growth (Fig. S3a). Mutants displaying altered biofilm growth in iron-supplemented medium were also examined for alterations in planktonic growth in normal or supplemented medium, to eliminate mutants with general fitness defects (Fig. S3a). Because we were seeking factors specifically involved in biofilm formation in excess iron, and not general biofilm factors, we performed a secondary biofilm assay to exclude mutants that displayed altered biofilm in both iron-supplemented and normal medium (Fig. S3b to g). The final E. faecalis transposon mutants that specifically altered biofilm formation in excess iron included single insertions in *phoH*, *ldh1*, *trxB2*, OG1RF_10589, and an intergenic region (Table S5). The predicted functions for all of the gene products were associated with metabolism functions and membrane transport.

10.1128/mBio.00626-17.3 FIG S3  Transposon library screen for mutants exhibiting changes in biofilm accumulation under iron supplementation. (a) Flow chart of screen process, including evaluation criteria for biofilm assays. (b to g) Secondary validation data for mutants passing primary evaluation. Light gray indicates normal medium, and dark gray indicates iron-supplemented medium. Secondary validation bar charts represent individual experiments and are separately presented, rather than being pooled, for greater clarity. Download FIG S3, PDF file, 0.2 MB. Copyright © 2018 Keogh et al. 2018 Keogh et al. This content is distributed under the terms of the Creative Commons Attribution 4.0 International license.

10.1128/mBio.00626-17.10 TABLE S5  E. faecalis genes involved in iron-induced biofilm growth. Download TABLE S5, DOCX file, 0.01 MB. Copyright © 2018 Keogh et al. 2018 Keogh et al. This content is distributed under the terms of the Creative Commons Attribution 4.0 International license.

### EET to iron occurs in E. faecalis biofilm.

**FIG 4:**
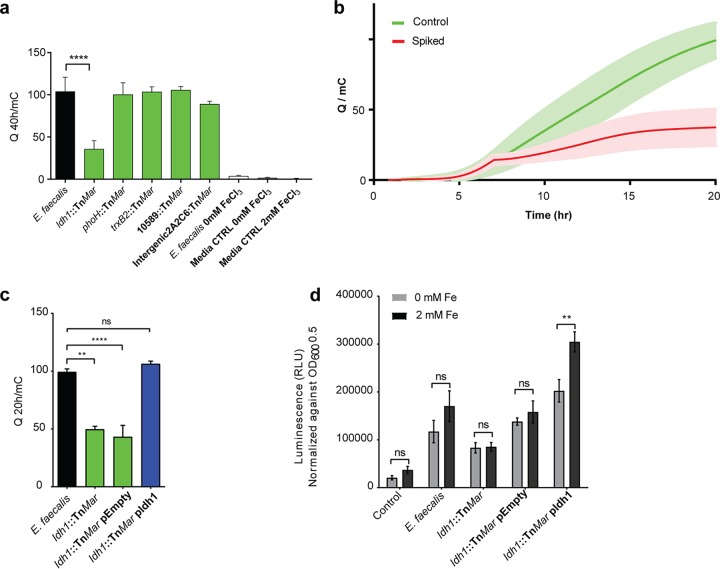
Extracellular electron transfer in E. faecalis biofilm. (a) Chronocoulometry current (*Q*) measurement, expressed in millicoulombs, of E. faecalis biofilm on a screen-printed electrode over 40 h in TSBG supplemented with 2 mM FeCl_3_ (supplemented). Abiotic controls and medium controls are indicated. Statistical significance was determined by one-way analysis of variance with Tukey’s test for multiple comparisons. *n* = 3 biological replicates. Error bars represent standard deviations from the mean. ****, *P* ≤ 0.0001. (b) Chronocoulometry of E. faecalis biofilm in iron-supplemented medium with a chelator spike (4 mM 2,2′-dipyridyl) at 7.5 h. Representative data from four independent experiments are shown, where the trend is consistent among all experiments. Statistical significance was determined by a paired two-tailed *t* test; error bars (light green or light red shading) represent standard deviations from the mean. ****, *P* ≤ 0.0001. (c) Chronocoulometry current (*Q*) measurement, expressed in millicoulombs, of E. faecalis wild-type, *ldh1* mutant, and complemented *ldh1* mutant biofilm on a screen-printed electrode over 40 h in TSBG supplemented with 2 mM FeCl_3_. Statistical significance was determined by one-way analysis of variance with Tukey’s test for multiple comparisons. *n* = 3 biological replicates. Error bars represent standard deviations from the mean. ****, *P* ≤ 0.0001. (d) ATP quantification within biofilm grown in TSBG and TSBG supplemented with 2 mM FeCl_3_ for 24 h. Nisin was included at 5 µg/ml, and erythromycin was included at 300 µg/ml for strains carrying a plasmid. Statistical significance was determined by one-way analysis of variance with Sidak’s test for multiple comparisons. *n* = 2 biological replicates. Error bars represent standard errors of the means. **, *P* ≤ 0.01; ns, not significant. ATP production was significantly increased for E. faecalis when grown in iron-supplemented medium in each of two individual experiments (*P* < 0.05) (data not shown).

Dissimilatory metal-reducing bacteria, such as *Shewanella* spp. and *Geobacter* spp., take advantage of extracellular redox-active metals as terminal electron acceptors for respiration by extracellular electron transfer (EET) (32, 33). *Shewanella* spp. and *Geobacter* spp. both have functional iron transport and regulation systems for intracellular iron homeostasis (34, 35). Electroactivity has also been detected in some *Enterococcus* spp. where exogenous redox mediators have been added as supplements (36, 37). Our data indicate that genes involved in energy production, redox control, and membrane transport contributed to iron-augmented biofilm (Table S2). We also observed iron deposits in the biofilm matrix that surrounds the bacterial cell surfaces of iron-enhanced biofilm (Fig. 2d, e, f, k, and m). We therefore hypothesized that the extracellular iron associated with E. faecalis biofilm matrix may function as electron sinks during biofilm metabolism, where Fe(III) is reduced to Fe(II) by E. faecalis. To test this, we used chronocoulometry to measure EET of E. faecalis biofilms grown in electrochemical cells on a carbon screen-printed electrode (SPE) maintained at high oxidative potential. This assay measures the integral of current over time, i.e., the charge transferred to the electrode over time, and therefore monitors in real time whether E. faecalis is capable of sustained EET in the presence of oxidized iron. Importantly, electrons are detected only at the electrode surface, and as such, any intracellular iron reduction cannot be measured by chronocoulometry. Chronocoulometry is commonly used to measure the electron transfer process in redox systems (38) and was previously used to measure EET in Pseudomonas aeruginosa (39). Using this assay, we observed that E. faecalis biofilms grew on the carbon electrode (Fig. S4a and b) and generated extracellular current in iron-supplemented medium, while all abiotic medium controls and E. faecalis biofilms grown in normal medium did not yield a current (Fig. 4a). Moreover, extracellular current generation was specific to the addition of iron, because we observed no current upon the addition of the other biofilm-augmenting metal magnesium, manganese, or heme (Fig. S4c). We then tested the five mutants identified through the transposon screen for the generation of extracellular current in the supplemented medium. Only the *ldh1* mutant displayed significantly reduced current compared to the parental strain (Fig. 4a), suggesting that l-lactate dehydrogenase (LDH) was involved in extracellular electron transfer in E. faecalis. We complemented the *ldh1* mutant using a nisin-inducible expression plasmid and restored biofilm formation to wild-type levels (Fig. S5). We quantified extracellular current by chronocoulometry for the *ldh1* mutant and the complemented strain and observed that current was also restored to the levels observed in the wild-type strain (Fig. 4c). To validate that extracellular iron was required, we spiked the electrochemical cell with the chelator 2,2′-dipyridyl to sequester iron. In the presence of iron chelation, we observed a significant reduction in current (Fig. 4b), demonstrating that soluble iron was instrumental to the process of EET. To ensure that chelator addition did not give rise to cell death leading to the observed loss of current production, we performed CLSM with live/dead staining to verify that chelation did not alter viability (data not shown). To test whether EET was generating increased energy for the bacterial biofilm, we quantified ATP levels at 24 h (Fig. 4d). We observed a nonsignificant increase in ATP in iron-supplemented medium for the wild-type strain, but not for the *ldh1* mutant, compared to normal medium. The *ldh1*-complemented strain, however, significantly increased ATP levels in iron-supplemented medium compared to the empty-vector control, suggesting that energy was more abundant in iron-supplemented biofilm when *ldh* was present.

10.1128/mBio.00626-17.4 FIG S4  Biofilm biomass and metal dependence of E. faecalis EET. (a and b) CLSM images of E. faecalis biofilm on a screen-printed electrode at the end of chronocoulometry measurements after 20 h of growth in normal TSBG (a) or TSBG supplemented with 2 mM FeCl_3_ (b). (c) Charge (millicoulombs) transferred from E. faecalis biofilm to the SPE after 20 h in normal TSBG or TSBG supplemented with 2 mM FeCl_3_, 2 mM manganese chloride (MnCl_2_), 2 mM magnesium sulfate (MgSO_4_), or 50 µM heme. These results were measured in glass electrochemical cells filled with 11 ml growth medium. All the other parameters are the same as described in Materials and Methods. The working electrode was poised at 200 mV versus the silver pseudoreference electrode of the SPE. Download FIG S4, PDF file, 0.4 MB. Copyright © 2018 Keogh et al. 2018 Keogh et al. This content is distributed under the terms of the Creative Commons Attribution 4.0 International license.

10.1128/mBio.00626-17.5 FIG S5  E. faecalis *ldh1* mutant biofilm growth under iron supplementation. E. faecalis biofilm growth at 120 h in TSBG and TSBG supplemented with 2 mM FeCl_3_. Nisin was included at 5 µg/ml for all samples, and erythromycin was included at 300 µg/ml for strains carrying a plasmid. *n* = 3 biological replicates. Statistical significance was determined by two-way analysis of variance with Sidak’s test for multiple comparisons. *n* = 3 with four technical replicates. *, *P* ≤ 0.05; **, *P* ≤ 0.01; ***, *P* ≤ 0.001; ****, *P* ≤ 0.0001. Error bars represent standard deviations from the means. Download FIG S5, TIF file, 1.6 MB. Copyright © 2018 Keogh et al. 2018 Keogh et al. This content is distributed under the terms of the Creative Commons Attribution 4.0 International license.

**FIG 5:**
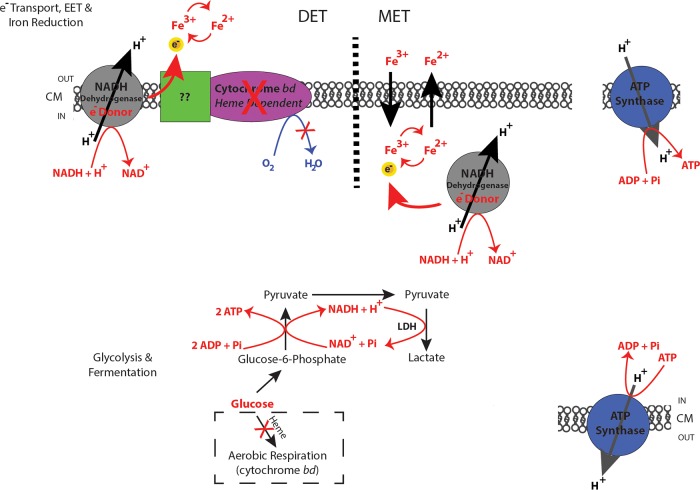
Model for fermentation and EET-dependent respiration metabolism in E. faecalis biofilm. Schematic model for E. faecalis biofilm metabolism describing EET through DET and MET mechanisms. ATP is generated by proton flow through membrane-integrated ATP synthases. Glycolysis and fermentation are required prior to EET-dependent respiration. This generates the fermentation end products required as the substrates for dehydrogenases acting as electron donors. DET occurs in the absence of heme, where extracellular iron can be reduced and thereby serve as an iron sink for electrons of the respiratory electron transport chain. MET occurs in the absence of heme, when iron is transported intracellularly and reduced, thereafter being exported and serving as an electron mediator/shuttle.

Taken together, our results allow us to propose several models for E. faecalis biofilm metabolism where EET, using biofilm matrix-associated iron, supports biofilm growth. In the first model (Fig. 5), we propose that in the absence of heme, as was the condition in this study, where cytochrome *bd* is nonfunctional for aerobic respiration, fermentation end products such as lactate can be used as the substrates by LDH to generate a modification of the electron transport chain. We suggest that this dehydrogenase may act as an electron donor transferring electrons to iron in the biofilm matrix and producing greater energy levels to augment biofilm growth. The precise route of electron flow is yet to be determined; however, we propose two possibilities based on data from our study and previous EET mechanism studies (40–47). LDH may act as an electron donor transferring electrons through the cytoplasmic membrane to iron acceptors in the biofilm matrix. As in other bacterial EET systems, direct electron transfer (DET) or mediated electron transfer (MET) mechanisms mediate the transit of electrons to the environment. For E. faecalis, this would require involvement of the membrane quinone pool (demethylmenaquinone [DMQ] specifically, since ubiquinone and menaquinone have not been identified in E. faecalis [48]) or membrane iron transporters to ultimately use iron in the biofilm matrix for EET and generate greater energy levels. Alternatively, LDH may not directly serve as an electron donor but may act indirectly to influence the expression or function of other mediators of electron transfer during EET.

## DISCUSSION

In this study, we characterize a new form of E. faecalis biofilm-specific metabolism where iron promotes EET, causing increased energy production and augmented biofilm growth. Although further investigation is needed to understand the physiological relevance of our results, understanding the diversity of metabolic possibilities may be important for understanding how E. faecalis colonizes different niches such as the human gastrointestinal tract, where iron is abundant (49), or the environment (50–52). The metabolic versatility of enterococci supports their survival in diverse and complex environments and communities, thus increasing the possibility of dissemination to new niches when iron is present (53).

Our findings demonstrate that E. faecalis is electroactive and likely takes advantage of Fe(III)/Fe(II) redox coupling to transfer electrons extracellularly, thus increasing energy production and augmenting biofilm growth. An Fe(III)/Fe(II) oxyhydroxide mixture is likely to represent a portion of the extracellular iron, which could serve to support biofilm growth by functioning as an electron sink (32). To explain this, we propose one model (Fig. 5) in which glycolysis and fermentation proceed prior to EET-dependent respiration to produce pyruvate. Pyruvate then oxidizes to lactate in the presence of LDH, resulting in the flow of electrons to an extracellular electron acceptor. EET can function by both DET, which involves outer membrane *c*-type cytochromes (42), and MET, which takes advantage of microbially produced redox mediators (39, 43, 54, 55). The precise site of EET-mediated iron reduction for E. faecalis, either intracellular or extracellular, is not yet known.

Bacteria capable of EET have been shown to mediate electron flow through membrane-embedded DMQ (56). While we did not detect a DMQ pathway mutant in our transposon library screen, this may be due to redundancies inherent in aerobic respiration chains or the substitution of enzyme functions required for DMQ-type molecules (57, 58). Alternatively, LDH could indirectly affect cellular chemistry, or other mediators of electron transfer to promote EET, energy production, and biofilm growth may be at play. Regardless, these findings extend the current knowledge regarding the extent of EET-capable bacterial species and environments, which are primarily viewed in the context of energy recovery in systems such as microbial fuel cells (33).

Gram-negative bacteria represent the majority of characterized EET producers, with a small number of Gram-positive bacteria, such as Thermincola potens and Desulfotomaculum reducens, characterized as being DET specific (59). Other Gram-positive microorganisms, such as *Corynebacterium* sp. (60) and E. faecalis itself, have been shown to produce electricity in microbial fuel cells when artificially supplied with the redox mediators anthraquinone-2,6-disulfonate (AQDS) and riboflavin (37), respectively. Enterococcus gallinarum can use iron as an electron acceptor, but the impact on growth was not determined (61). Our work demonstrates that E. faecalis achieves augmented growth through a biofilm-specific iron-dependent EET metabolism, and we show that LDH contributes to this phenotype.

E. faecalis and other lactic acid bacteria (LAB) encode the components necessary for aerobic respiration metabolism (62). These are (i) NADH dehydrogenases functioning as electron donors and (ii) a quinone pool to transfer electrons to (iii) the terminal electron acceptor complex cytochrome oxidase (63). The E. faecalis cytochrome *bd* enzyme can use oxygen as a terminal electron acceptor only when exogenous sources of its cofactor heme are available (5, 64). E. faecalis does not synthesize porphyrin and, in the absence of heme, relies on fermentation. Our experimental assays mimic the absence of heme because it is not present in the growth medium. Our genetic and bioelectrochemical data suggest that E. faecalis LDH contributes to EET, in which electrons are transferred to biofilm matrix-associated iron sinks. Dehydrogenases transfer hydride from one substrate to another in a reversible reaction that relies on the interconversion of NADH and NAD^+^. Previous studies suggest that, in contrast to most bacterial species that rely on the tricarboxylic acid (TCA) cycle to produce NADH, LAB instead rely on fermentation for its production (65). Fermentation therefore is a necessary metabolic phase prior to the use of EET to support biofilm metabolism. In Lactococcus lactis, a fermenting LAB strain, activation of respiration metabolism in the absence of heme and oxygen reduction occurs by copper reduction via the menaquinone pool (66). This supports our finding that extracellular iron is functioning as an electron acceptor and/or mediator that can activate respiration metabolism in the absence of heme and cytochrome *bd* activity.

Our biofilm data demonstrate an iron-specific E. faecalis enhanced biofilm growth response, with only FeCl_3_, heme, FeSO_4_, and Fe_2_(SO_4_)_3_ stimulating the response. Heme availability enables respiration through cytochrome *bd* activity in the presence of oxygen (29). FeSO_4_ and Fe_2_(SO_4_)_3_ are more labile to oxidative changes and precipitation, required for metabolically driven iron reduction, than the more soluble ferric citrate, which does not enhance biofilm growth, suggesting that the mechanism governing enhanced biofilm growth is most specific to FeCl_3_.

E. faecalis can flourish in iron-limited environments and tolerate oxidative stress which can be induced by a high-iron environment, contrasting with many other bacterial species where iron is an essential growth nutrient and requires strict iron homeostasis (67). Nonetheless, our ICP-MS analysis shows that iron represents a large proportion of the E. faecalis metallome under normal conditions. The modulation of the E. faecalis metallome with changes in conditions of either iron limitation or abundance suggests alternative roles for this metal. The absence of detectable iron in the E. faecalis mutants with disruptions in genes predicted to encode iron transport and regulatory components, analyzed by ICP-MS under iron limitation, suggests that these gene products function in iron acquisition. Previous transcriptomic studies of planktonic cultures propose roles for E. faecalis transport systems in iron acquisition; however, our ICP-MS analysis is the first functional characterization of these systems (11, 12). Manganese has been reported to substitute for iron as a cofactor in essential cellular reactions of LAB species; however, our data demonstrate that iron represents a greater proportion of the E. faecalis metallome than manganese under normal and iron-supplemented conditions (7, 8). The ICP-MS analysis equally highlights that a number of other metals, such as zinc, potassium, and magnesium, are abundant. Cobalt and zinc are important metals for bacterial growth, and we have identified a number of genes governing cobalt and zinc utilization in E. faecalis (68). While the intracellular iron levels in the iron-supplemented medium were greater than that under the normal condition, no iron transport systems could be identified under these conditions. However, overlapping functionality in nutrient transport is a common strategy for bacteria, and so single-gene mutations may not be sufficient under this condition. Intracellular storage of iron may be strategic in anticipation of acceptor-limited conditions under which this metal can be deposited extracellularly.

Colonization and persistence in host niches and the environment require adaptability in the exploitation of available resources for energy production and growth. Our work highlights a new form of metabolism where, in the absence of heme, components of respiration can be utilized for EET using extracellular iron. This deeper understanding of mechanisms governing biofilm metabolism in humans and clinically relevant bacterial species will enhance approaches to modify or eradicate these reservoirs.

## MATERIALS AND METHODS

### Bacterial strains and growth conditions.

Enterococcus faecalis OG1RF (ATCC 47077) (69) was grown in brain heart infusion broth (BHI) and cultured at 37°C under static conditions. E. faecalis SD234 is an OG1RF strain derivative harboring a constitutively expressed *gfp* gene (70). Overnight cultures were normalized to 2 × 10^8^ to 4 × 10^8^ CFU/ml in phosphate-buffered saline (PBS), equivalent to an optical density at 600 nm (OD_600_) of 0.7 for E. faecalis. For planktonic and biofilm assays, bacteria were cultured at 37°C (under 200-rpm orbital shaking or static conditions, respectively) with tryptone soy broth supplemented with 10 mM glucose (TSBG) and solidified with 1.5% agar when appropriate (Oxoid Technical no. 3). Planktonic assay mixtures were inoculated from the normalized stocks to a starting CFU of 2 × 10^5^ to 4 × 10^5^ CFU/ml. E. faecalis strains harboring pMSP3535 (source, Gary M. Dunny; Addgene plasmid 46886) or pMSP3535::*ldh1* (source, Axel Hartke [71]) were selected for with 300 µg/ml erythromycin and induced with 5-µg/ml nisin-supplemented BHI. BHI was supplied by Becton, Dickinson and Company, Franklin Lakes, NJ. TSB and agar were supplied by Oxoid Inc., Ontario, Canada. Metals for supplementation were added during medium preparation. Ferric citrate hydrate (≥98%); magnesium chloride, anhydrous (≥98%); copper chloride dihydrate (≥99%); ferrous sulfate heptahydrate (≥99%); ferric sulfate hydrate (≥97%); ferric chloride, anhydrous (≥99%); heme (≥90%); and the chelator 2,2′-dipyridyl (≥99%) were all supplied by Sigma-Aldrich, St. Louis, MO, USA. Manganese chloride tetrahydrate and zinc chloride were supplied by Merck Millipore (Singapore).

### Biofilm assay.

Bacterial cultures were normalized as described above and inoculated at 1.6 × 10^6^ to 3.2 × 10^6^ CFU/200-µl microtiter well in TSBG in a 96-well flat-bottom transparent microtiter plate (Thermo Scientific, Waltham, MA) and incubated at 37°C under static conditions. Uninoculated medium controls supplemented with metals to the highest concentration relevant to the assay were included to check for supplemented metal precipitation. Supernatants were discarded, and the microtiter plate was washed twice with PBS. To stain surface-adherent bacteria, 200 μl of crystal violet solution at 0.1% (wt/vol) (Sigma-Aldrich, St. Louis, MO, USA) was added to each well and incubated at 4°C for 30 min. This solution was discarded, and the microtiter plate was washed twice with PBS followed by crystal violet solubilization with 200 μl per well ethanol-acetone (4:1) for 45 min at room temperature. The intensity of crystal violet staining was measured by absorbance at 595 nm (OD_595_) using a Tecan Infinite 200 Pro spectrophotometer (Tecan Group Ltd., Männedorf, Switzerland).

### Flow cell biofilm assay.

Flow cell biofilm studies were performed as previously described with minor modifications (72). Bacterial cultures were normalized to 2 × 10^6^ to 4 × 10^6^ CFU/ml in PBS, and 250 μl of this stock was injected through the Stovall flow cell system inlet silicon tube connected to the flow cell chamber. This inoculation was performed when the system flow was halted by clamping both the inlet and outlet silicon tubes. The chamber was inverted for 1 h to facilitate bacterial adherence to the glass slide surface. The flow cell system was then reset and unclamped, and the medium feed was set to 4.5 ml/h.

### Transposon library screen.

The cryogenically stocked, 96-well-format E. faecalis OG1RF mariner transposon library consisted of 14,978 individual mutants (31). This library was cultured using a cryoreplicator (Adolf Kühner AG) to inoculate DeepWell blocks (Greiner Bio-One) containing 1 ml BHI medium for overnight incubation at 37°C with shaking at 220 rpm. Cultures were normalized to an OD_600_ of 0.1 (2 × 10^8^ to 4 × 10^8^ CFU/ml) in PBS with the Tecan Infinite 200 Pro spectrophotometer (Tecan Group Ltd., Switzerland) using a 96-well microtiter plate. The primary screen of the library was performed by inoculating a microtiter well with 1.6 × 10^6^ to 3.2 × 10^6^ CFU/200 µl in TSBG medium supplemented with 2 mM FeCl_3_. The microtiter plates were then incubated at 37°C, statically, inside a moistened chamber to prevent evaporation of medium. Biofilm was quantified by crystal violet staining as described above. Mutants with either reduction or further enhancement of biofilm signal compared to wild-type controls were then validated using two independent biological replicates for each mutant in TSBG biofilm assays. This primary validation was followed by a planktonic growth validation in TSBG and TSBG medium supplemented with 2 mM FeCl_3_. A secondary validation using three independent biological replicates for each mutant was performed in biofilm assays with TSBG and TSBG medium supplemented with 2 mM FeCl_3_ to eliminate any mutants exhibiting defects in biofilm formation under normal conditions. Mutants harboring multiple transposon (Tn) insertions were excluded from the screen.

### Mapping Tn insertions.

Genomic DNA (gDNA) was extracted using the Wizard genomic DNA purification kit (Promega) from transposon mutants that were not originally mapped. The gDNA was quantified and assessed for nucleic acid quality by the Qubit highly sensitive double-stranded DNA (dsDNA) assay (Invitrogen) and a NanoDrop spectrophotometer. Sequencing was performed using an Illumina MiSeq sequencer. *De novo* reads were assembled using CLC Genomics Workbench version 8.0 and E. faecalis OG1RF as a template. The Tn insertion site was identified by BLAST using the mariner transposon sequence and identification of the flanking genomic sequence. Transposon mutant strains were named to include library location information, gene name, or intergenic information, followed by “TnMar.” For example, (4.2A1 F1)10589:TnMar indicates the transposon mutant for OG1RF_10589 located in block 4.2A1 of the library in position F1 of the microtiter plate.

### Electrochemical setup and analysis.

Screen-printed electrodes (SPEs) (model DRP-C110; DropSens, Spain) consisting of a carbon working electrode, carbon counter electrode, and Ag pseudoreference electrode were controlled by a multichannel potentiostat (VSP; Bio-Logic, France) in an electrochemical cell of 9-ml working volume sealed with a Teflon cap. Chronocoulometry was used to characterize the electrochemical activity of live microbial cultures by measuring the charge *Q* passed over the course of growth, with *Q* (millicoulombs) at 40 h used for comparison. During chronocoulometry, the working electrode was maintained at 200 mV versus the Ag pseudoreference electrode of the SPE. This potential was chosen as it is high enough to reoxidize Fe(II) to Fe(III) while being low enough to avoid damaging the bacteria. By applying a set potential to an electrode acting as an electron acceptor, the extracellular current generated can be tracked with time, the integral of which represents the total charge passed. By looking at the charge passed at a fixed time, the electroactivity of a culture/mutant, etc., can be established. Bacterial stocks of 2 × 10^8^ to 4 × 10^8^ CFU/ml for electrochemical experiments were prepared as described above, and electrochemical cells were inoculated to 2 × 10^5^ to 4 × 10^5^ CFU/ml. All electrochemical experiments were conducted at 37°C using TSBG medium supplemented with 2 mM FeCl_3_ unless otherwise stated. The iron chelator 2,2′-dipyridyl (Sigma-Aldrich, USA) was spiked in selected experiments to quench EET.

### Electrode biofilm biomass quantification.

Biofilm biomass was analyzed by CLSM directly from screen-printed electrodes with 5 individual z-stack images per biological replicate. Images were acquired using an LSM 780 confocal microscope (Zeiss, Germany) equipped with a 20×/0.8 Plan-Apochromat objective. Viability staining was performed using 5 ml of 200 µM propidium iodide (PI) and 33.4 µM SYTO 9 nucleic acid incubated in the dark for 15 min. After staining, the screen-printed electrodes were washed with 5 ml of 1× phosphate-buffered saline (PBS) twice, mounted inverted on the glass slide, and viewed using an LSM 780 confocal microscope. Imaging of propidium iodide-stained cells was performed using excitation/emission wavelengths at 561 nm and 633 nm, respectively, while SYTO9 nucleic acid-stained cells were imaged using excitation/emission wavelengths at 488 nm and 500 nm, respectively. Image analysis was performed using Carl Zeiss microimaging software. Analysis of biomass was performed using Imaris software.

### Thin-section transmission electron microscopy (TEM).

Biofilms were grown using the flow cell protocol or the standard biofilm protocol described above, but with the latter using a 6-well plate. The flow cell biofilm was resuspended in a 2% paraformaldehyde-2.5% glutaraldehyde solution (Polysciences, Warrington, PA) in 100 mM PBS (pH 7.4) for 1 h at room temperature. The samples were then embedded in 2% low-melting-point agarose, washed in PBS, and postfixed in 1% osmium tetroxide for 1 h. Samples were rinsed extensively in distilled water (dH_2_O) prior to en bloc staining with 1% aqueous uranyl acetate (Ted Pella, Inc., Redding, CA) for 1 h. Following several rinses in dH_2_O, samples were dehydrated in a graded series of ethanol and embedded in Eponate 12 resin (Ted Pella, Inc., Redding, CA). Sections of 95 nm were cut with a Leica Ultracut UCT ultramicrotome (Leica Microsystems, Inc., Bannockburn, IL, USA), stained with uranyl acetate and lead citrate, and viewed on a JEOL 1200 EX transmission electron microscope (JEOL USA, Inc., Peabody, MA).

### Energy-dispersive X-ray spectroscopy (EDS) TEM.

Samples were prepared as described above for TEM and viewed on an aberration-corrected JEOL ARM 200 cold field-emission gun transmission electron microscope in high-angle annular dark-field scanning transmission (HAADF STEM) mode using spot size 6. The sample was held in a JEOL Be double tilt holder tilted by about 12° toward the X-ray detector. STEM images were collected using a JEOL annular dark-field detector on a Gatan Digiscan with 2,048 by 2,048 pixels and a dwell time of 10 µs. Energy-dispersive X-ray maps were collected using an Oxford Aztec system with an 0.7-sr collection solid angle by scanning the beam with multiple frames over a period of about 5 min at a resolution of 512 by 512 pixels.

### ATP quantification.

Biofilms were grown in 6-well microtiter plates at 37°C for 24 h under static conditions as described above. Spent medium was removed, and the adherent biofilm was washed with 1 ml of 1× PBS, disrupted into a single-cell suspension, and normalized to an OD of 0.5. One hundred microliters of normalized biofilm sample was added to 100 µl of prepared BacTiter-Glo reagent from the BacTiter-Glo microbial cell viability assay kit (Promega) and incubated at room temperature for 5 min before measuring luminescence using a Tecan Infinite 200 Pro spectrophotometer (Tecan Group Ltd., Männedorf, Switzerland). An integration time of 0.25 to 1 s per well was used.

### Confocal laser scanning microscopy and 3D reconstruction.

Biofilm morphology, biomass, and cell distribution were analyzed by CLSM directly from flow cell chamber glass microscope slides at three separate locations (inlet, middle, and outlet areas) with 3 individual z-stack images per technical replicate. Images were acquired using an LSM 780 confocal microscope (Zeiss, Germany) equipped with a 20×/0.8 Plan-Apochromat objective and controlled by ZEN software. Samples were illuminated with a 488-nm argon laser line, and the GFP emitted fluorescence was collected in the 507- to 535-nm range. Optical sections (425 by 425 µm) were collected every 5 µm through the entire biofilm thickness, and the signal from each section was averaged 2 to 4 times. Fiji software (73) was used for further processing (level adjustment and stack reslice). For the 3D biofilm reconstructions, optical slices (85 by 85 µm) were acquired with a 63×/1.4 Plan-Apochromat oil immersion lens every 0.3 µm through the entire biofilm thickness or until loss of the fluorescence signal (due to light scattering, absorption, and possible fluorescence quenching of thick iron-supplemented biofilms). The center of mass for each cell in 3D space was found using MosaicSuite for Fiji (74), and then coordinates were filtered in R (75). To visualize the biofilm matrix and spatial organization, coordinates were plotted as spheres with cell size diameter and color-coded *z*-depth.

### ICP-MS.

E. faecalis cultures were prepared as previously described with minor modifications (76). Overnight cultures were normalized as described above, and 1 × 10^5^ to 2 × 10^5^ CFU/well was inoculated into DeepWell blocks (Greiner Bio-One) containing 2 ml TSBG medium and incubated overnight at 37°C under static conditions. Three biological replicates with five technical replicates were prepared for each E. faecalis strain, and following incubation, the technical replicates were pooled prior to preparation for ICP-MS. Harvested cell pellets were washed with 10 mM EDTA (Ambion Thermo Fisher Scientific, USA) prepared in liquid chromatography-mass spectrometry (LC-MS)-grade dH_2_O (Sigma-Aldrich, St. Louis, MO, USA) and washed three times with LC-MS-grade dH_2_O. Cells were then concentrated to 2 × 10^8^ to 4 × 10^8^ CFU/ml in LC-MS-grade dH_2_O. Each cell pellet was digested in 500 µl of 69% nitric acid and 250 µl of 31% hydrogen peroxide. Following sample digestion, all the samples were diluted with LC-MS-grade dH_2_O to a 2% (wt/vol) nitric acid concentration in the final solution.

An Agilent 7700 series model ICP-MS system (Agilent, Santa Clara, CA) was used for simultaneous determination of selected elements (Mg, Al, P, K, Ca, V, Cr, Mn, Fe, Co, Ni, Cu, and Zn) in prepared E. faecalis samples. Prior to sample measurement and quantification, stock solutions of a multielement calibration standard (Inorganic Ventures, VA, USA) were serially diluted (0 μg/liter to 1,000 μg/liter) and run on the system. For each measurement (standards, samples, blanks, and quality controls), addition of internal standard (Sc; 100 mg/liter, Agilent, USA) was performed to correct for physical matrix effects. Blanks were determined together with samples for every run, and the mean for three runs was determined for each sample. Full quantitative analysis was performed against calibration standards for each element. Quality control samples (multielement calibration solution; 100 μl) were inserted and run at regular intervals during the experiment to ensure reliability of the data and to eliminate signal drift or interference.

For statistical analysis, 10 metals were measured for 22 phenotypes (E. faecalis OG1RF wild type and 21 mutants) under three nutrient conditions, with 3 biological replicates for each mutant and under each condition. During the ICP-MS run, 3 technical replicates were measured from each sample, which were then averaged and used for statistical analysis. The concentration of the metals in a blank control (500 µl of 69% nitric acid and 250 µl of 31% hydrogen peroxide) was subtracted from the concentration of metals in the samples. Metals whose levels were below the detection limit were coded as not detected. For statistical analysis, metal concentrations were normalized using log base 10 transformation. To test differences between the metal levels in mutants and the control under each nutrient condition, Welch’s two-sample *t* tests were performed in R using the t.test function, with correction for multiple comparisons made using the Benjamini-Hochberg procedure.
