# Correction for Keogh et al., “Extracellular Electron Transfer Powers Enterococcus faecalis Biofilm Metabolism”

**DOI:** 10.1128/mBio.01080-19

**Published:** 2019-06-18

**Authors:** Damien Keogh, Ling Ning Lam, Lucinda E. Doyle, Artur Matysik, Shruti Pavagadhi, Shivshankar Umashankar, Pui Man Low, Jennifer L. Dale, Yiyang Song, Sean Pin Ng, Chris B. Boothroyd, Gary M. Dunny, Sanjay Swarup, Rohan B. H. Williams, Enrico Marsili, Kimberly A. Kline

**Affiliations:** a Singapore Centre for Environmental Life Science Engineering, Nanyang Technological University, Singapore; b School of Biological Sciences, Nanyang Technological University, Singapore; c Interdisciplinary Graduate School, Nanyang Technological University, Singapore; d Singapore Centre for Environmental Life Science Engineering, National University of Singapore, Singapore; e Department of Microbiology, University of Minnesota Medical School, Minneapolis, Minnesota, USA; f Singapore Phenome Center, Lee Kong Chian School of Medicine, Nanyang Technological University, Singapore; g School of Materials Science and Engineering, Nanyang Technological University, Singapore; h Department of Biological Sciences, National University of Singapore, Singapore; i School of Chemical and Biomedical Engineering, Nanyang Technological University, Singapore

## AUTHOR CORRECTION

Volume 9, issue 2, e00626-17, 2018, https://doi.org/10.1128/mBio.00626-17. The following funding information and acknowledgment should have appeared in the article.

Artur Matysik was supported by the National Medical Research Council under its Clinical Basic Research Grant (NMRC/CBRG/0086/2015).

