# Supplementary figures and images for: Extracellular Electron Transfer Powers Enterococcus faecalis Biofilm Metabolism
Source: mBio. 2018 Apr 10;9(2):e00626-17. doi: 10.1128/mBio.00626-17 (PMC5893876; doi:10.1128/mBio.00626-17)

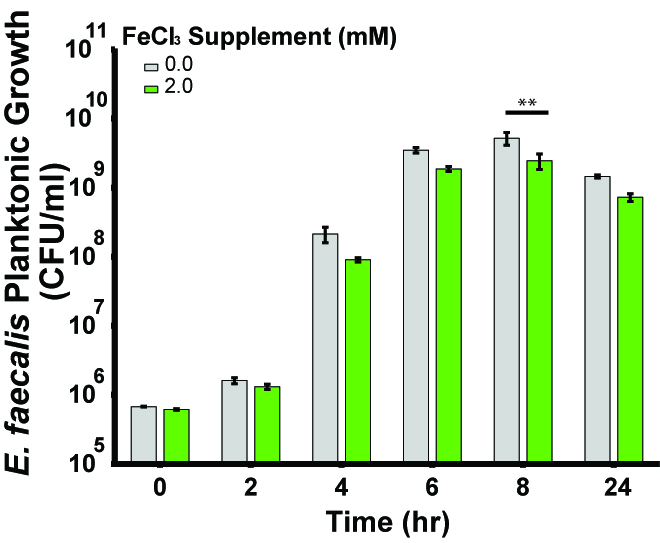

Supplement: FIG S1 [file mbo002183826sf1.tif]

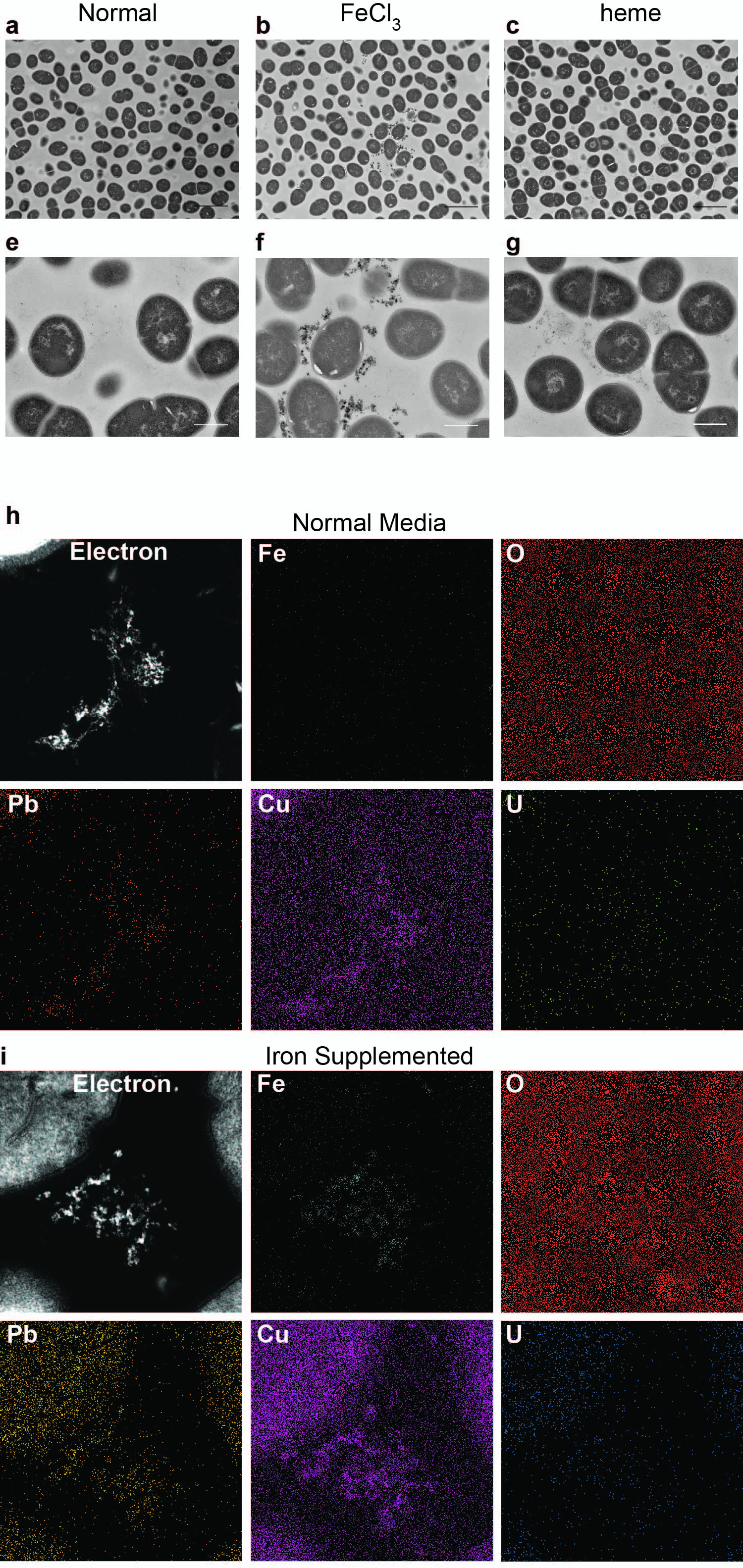

Supplement: FIG S2 [file mbo002183826sf2.jpg]

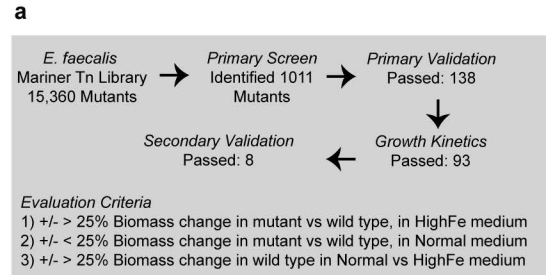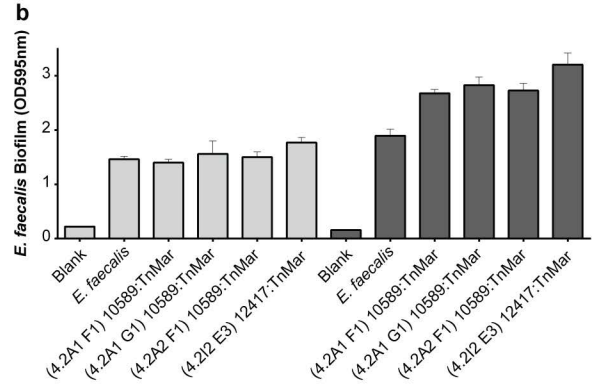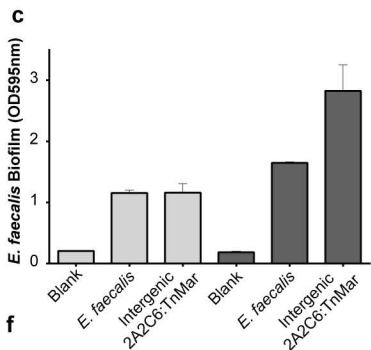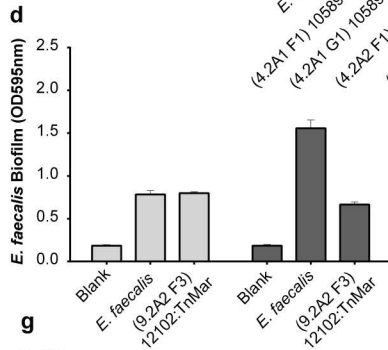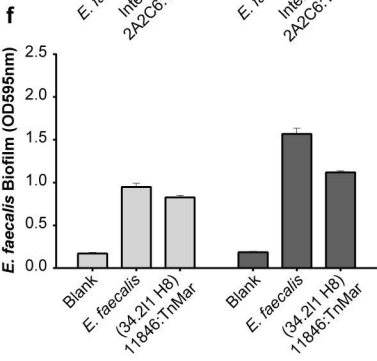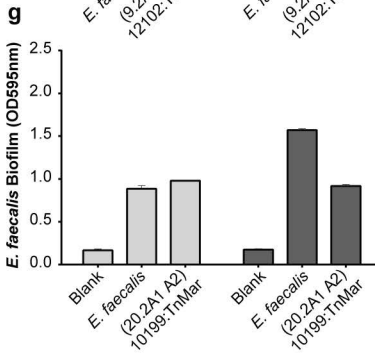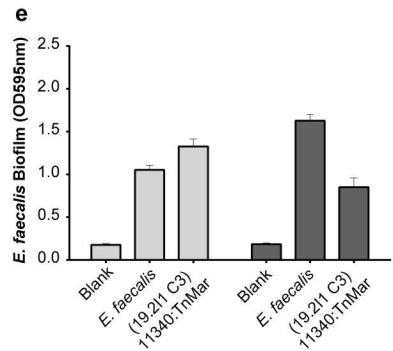

Supplement: FIG S3 [file mbo002183826sf3.pdf]

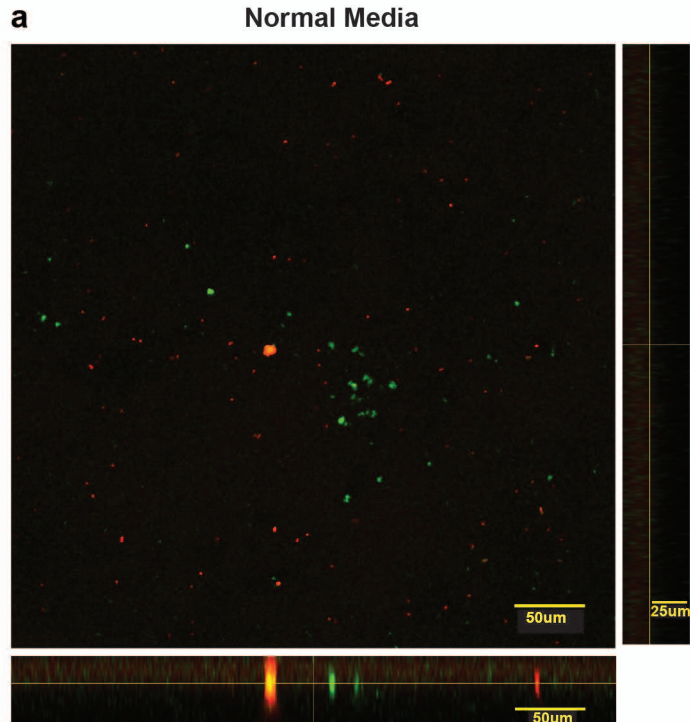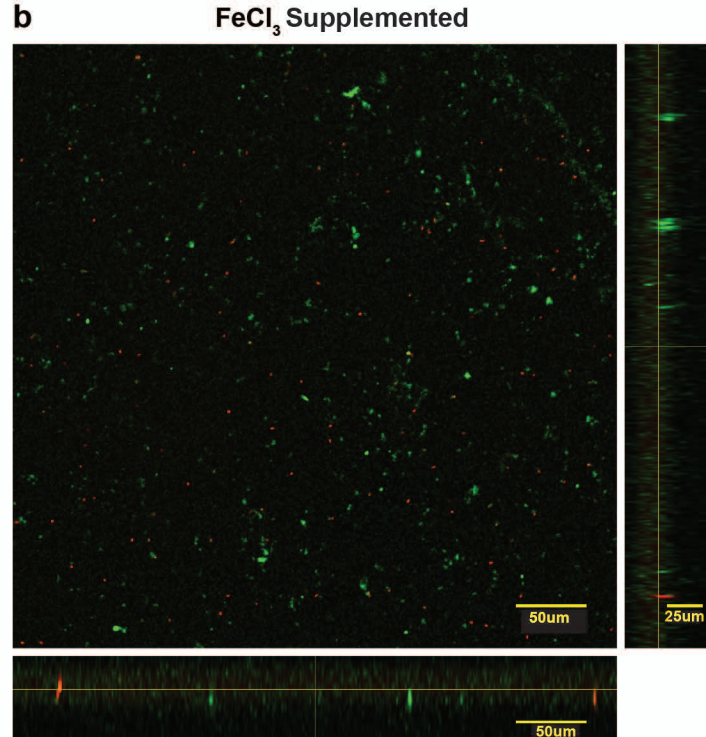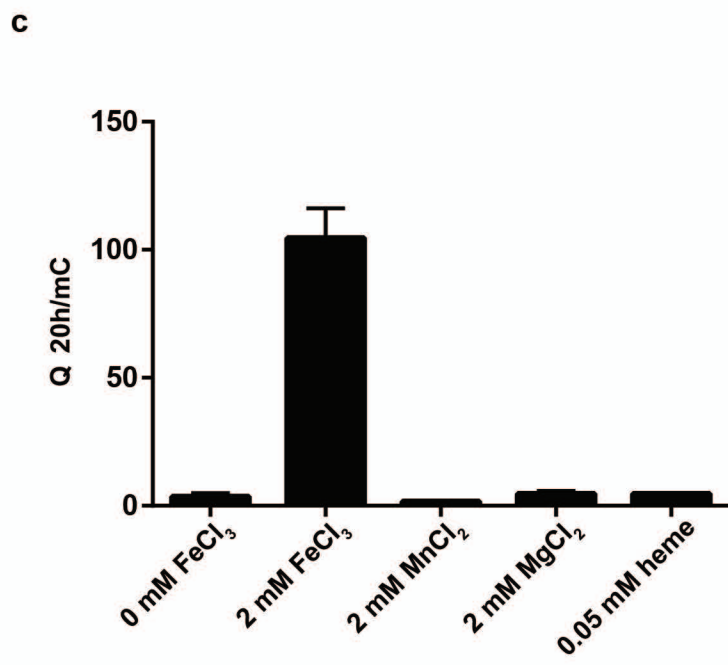

Supplement: FIG S4 [file mbo002183826sf4.pdf]

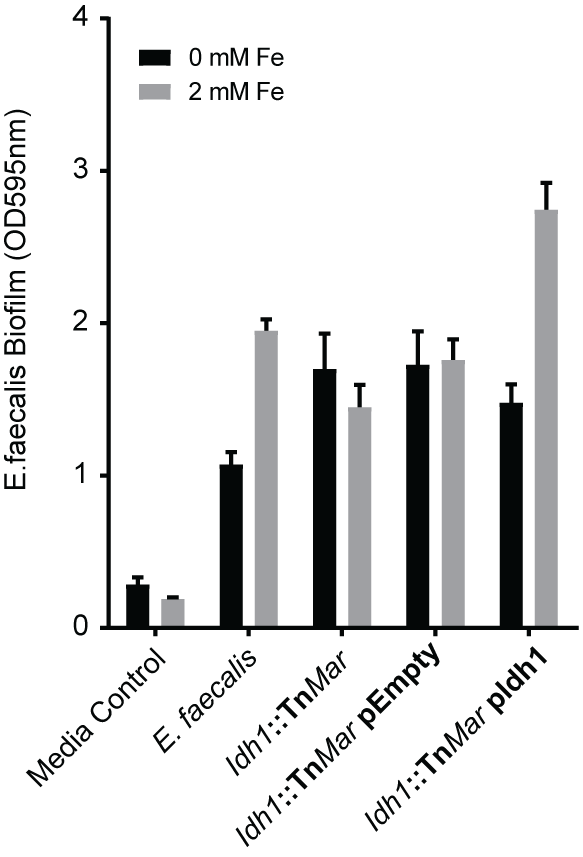

Supplement: FIG S5 [file mbo002183826sf5.tif]
