# Supplementary material for: Extracellular Electron Transfer Powers Enterococcus faecalis Biofilm Metabolism
Source: mBio. 2018 Apr 10;9(2):e00626-17. doi: 10.1128/mBio.00626-17 (PMC5893876; doi:10.1128/mBio.00626-17)
Supplement: TABLE S5 [file mbo002183826st5.docx]

**Table S5: *E. faecalis* genes involved in iron-induced biofilm growth**

| **Mutant ID** | **Gene Locus / Name** | **Intergenic Location** | **Predicted/Annotated Function** | **Biofilm Phenotype*** | **Number of Hits** | **Description** |
| --- | --- | --- | --- | --- | --- | --- |
| Intergenic2A2C6:TnMar | n.a. | 1427310-1427493 | Between OG1RF_11362 & OG1RF_11363 | Enhanced (+71.0%) | 1 | n.a. |
| 10589:TnMar | OG1RF_10589 | n.a. | Cation efflux protein | Enhanced (+37.0%) | 3 | Cation transporter |
| 12102:TnMar | OG1RF_12102 / *trxB2* | n.a. | Thioredoxin-disulfide reductase | Reduced (-60.0%) | 1 | NADH-dependent, Redox control |
| 10199:TnMar | OG1RF_10199 / *ldh1* | n.a. | L-lactate dehydrogenase | Reduced (-39.5%) | 1 | NADH-dependent |
| 11846:TnMar | OG1RF_11846 / *phoH* | n.a. | PhoH family protein | Reduced (-28.0%) | 1 | Induced by phosphate starvation |

Notes: n.a. = not applicable; * = mutant compared to wild type biofilm in the iron supplemented medium
